# Redefining Molecular Probes for Monitoring Subcellular Environment: A Perspective

**DOI:** 10.1021/acs.analchem.4c05022

**Published:** 2024-11-22

**Authors:** Santiago García, Gustavo Carmona-Santiago, Arturo Jiménez-Sánchez

**Affiliations:** Instituto de Química, Universidad Nacional Autónoma de México, Ciudad Universitaria, Circuito Exterior s/n, Coyoacán, Ciudad de México 04510, México

## Abstract

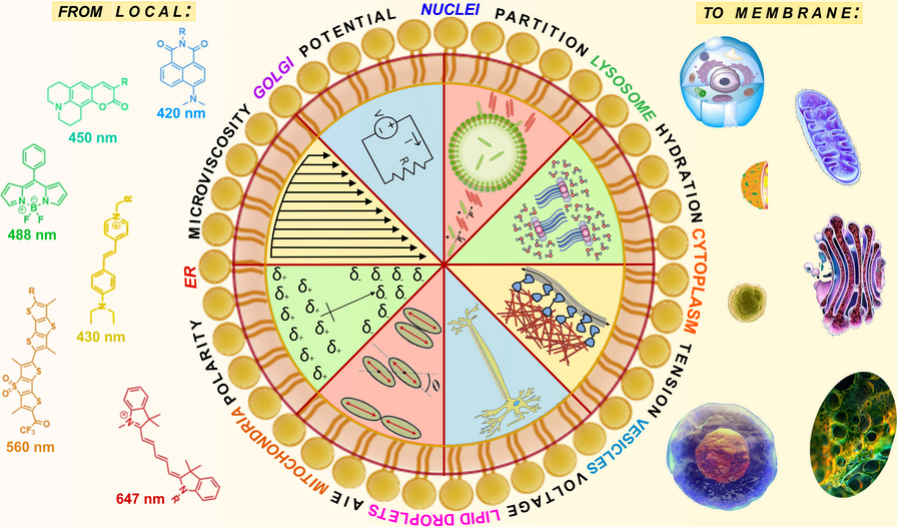

The development of small-molecule fluorescent probes has revolutionized the monitoring of *in vivo* physicochemical parameters, offering unprecedented insights into biological processes. In this perspective, we critically examine recent advances and trends in the design and application of fluorescent probes for real-time *in vivo* monitoring of subcellular environments. Traditional concepts such as membrane potential, microviscosity, and micropolarity have been superseded by more biologically relevant parameters like membrane voltage, tension, and hydration, enhancing the accuracy of physiological assessments. This redefinition not only presents an evolved concept with broader applications in monitoring subcellular dynamics but also addresses the unmet needs of subcellular biology more effectively. We also highlight the limitations of commonly used probes in providing specific information about the redox environment, noting their nonspecificity to oxidants and the influence of various chemical interactions. These probes typically rely on free radical mechanisms and require metal catalysts to react with hydrogen peroxide. They include naphthalimide, fluorescein, BODIPY, rhodamine, cyanine cores to cover the UV–vis–near-infrared window. The motif of this perspective is to provide critical insights into trending fluorescent-based systems employed in real-time or *in vivo* physicochemical-responsive monitoring, thus aiming to inform and inspire further research in creating robust and efficient fluorescent probes for comprehensive *in vivo* monitoring applications.

## Introduction

Fluorescent molecular probes have emerged as indispensable tools for exploring both biological and chemical realms.^1^ This surge is propelled by advancements in optical instrumentation, which now enables the detection of single molecules on a femtosecond time scale and super-resolution imaging techniques,^2^ facilitating subcellular dynamics imaging with unprecedented nanoscopic precision.^3^ Simultaneously, organic chemistry has witnessed rapid progress, yielding a plethora of intricate molecules, including sophisticated fluorescent probes.^4−6^ These probes play a pivotal role in elucidating the structural, compositional, and interactive dynamics of biological systems through their fluorescence signals,^7,8^ focusing on naphthalimide, fluorescein, BODIPY, rhodamine, cyanine cores designed for the blue (DAPI, λ_exc_ = 420 nm, λ_em_ = 460 nm), green (GFP, λ_exc_ = 488 nm, λ_em_ = 500 nm), orange (TxR, λ_exc_ = 560 nm, λ_em_ = 610 nm) and red (Cy5, λ_exc_ = 647 nm, λ_em_ = 700 nm) confocal channels.

A precise definition of a molecular probe for biosensing comprises at least two essential components: a biological affinity element and a reporter moiety.^9^ Together, these elements function to detect specific chemicals or biomolecules. *In vivo* biosensing holds the promise of transforming healthcare through personalized medicine.^10^ By monitoring a health baseline of an individual in real-time, even minor deviations from normal signals could serve as early indicators of potential health issues.^11−13^ This capability not only enhances preventive care but also offers continuous therapeutic drug monitoring, ensuring precise dosing tailored to each patient unique pharmacokinetics. Despite numerous molecular probes detecting physiologically relevant analytes, and their potential for *in vivo* monitoring, few have advanced beyond preclinical animal studies or gained approval for human implantation.^14,15^ This underscores the gap between promising research and practical application in healthcare.

Despite advancements in fluorescent probes for subcellular monitoring, a key gap remains in capturing real-time cellular complexities, especially under stress or disease conditions. Existing probes, while valuable, often fail to capture essential biophysical parameters like membrane tension and hydration. Traditional probes often fail to capture rapid, transient changes in subcellular environments, limiting insights into cellular responses. In this regard, organelle translocation probes offer a promising solution.^16−18^ This perspective aims to bridge that gap by redefining probe design paradigms to incorporate biologically relevant parameters. We aim to inspire a new generation of probes for more precise assessments of subcellular environments, opening pathways for research in cellular dynamics and biomedical applications.

## Redefining Local Microviscosity Probes for Advanced Cellular Sensing

**Figure 1:**
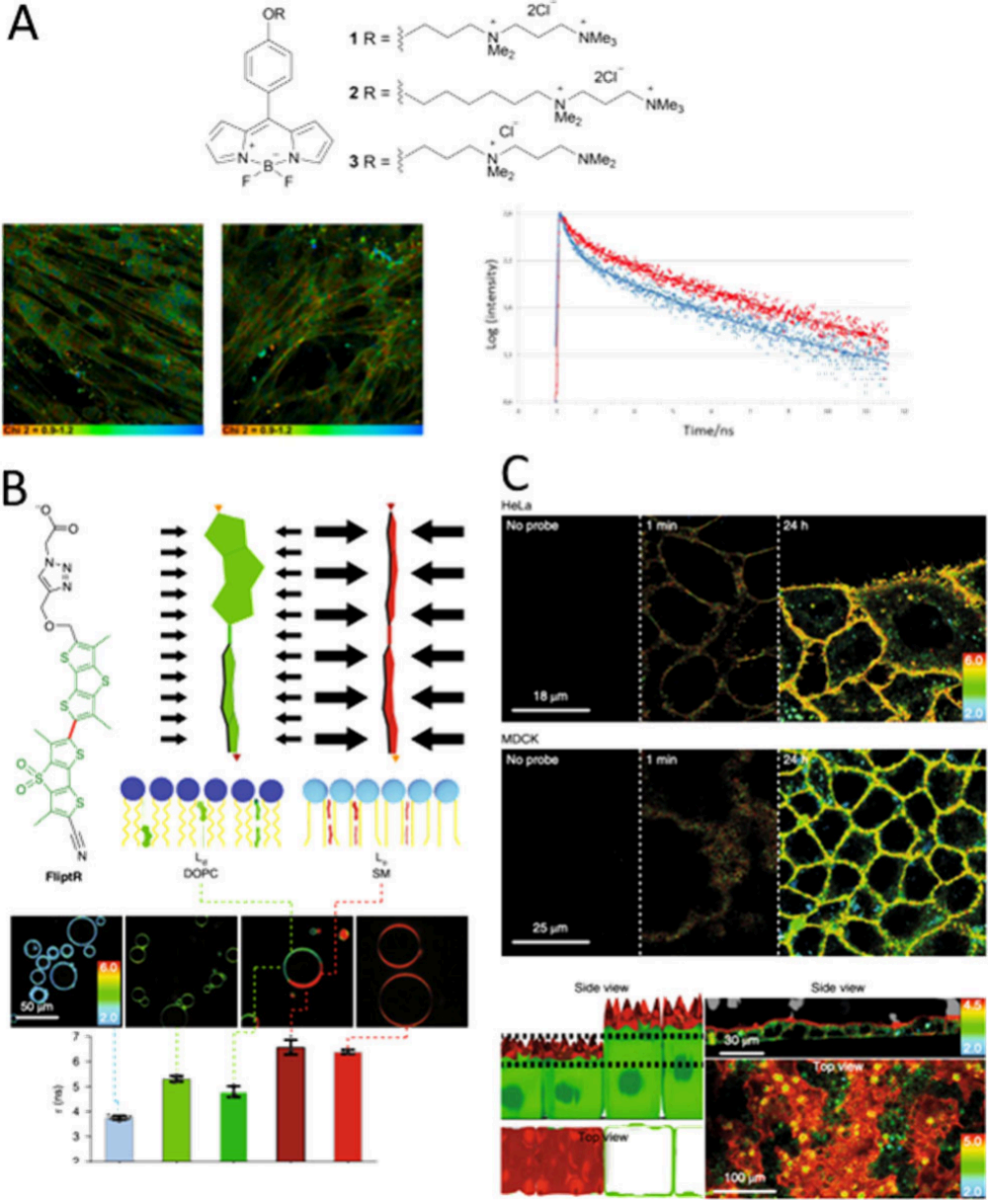
Redefining local polarity for precise membrane healthy status monitoring, used for GFP, λ_exc_ = 488 nm and red Cy5, λ_exc_ = 647 nm channels. (A) Molecular structure of the BODIPY-derived viscosity probes **1**-**3**. (B) Illustration of the proposed flipper ground-state planarization model and corresponding *in vitro* imaging of cells and DOPC vesicles. (C) Relationship between TCSPC spectroscopy and FLIM microscopy for lifetime correlation analysis.

Viscosity is a macroscopic property, making it challenging to obtain absolute values in a medium using fluorescent probes. Consequently, the term “microviscosity” is often used, though it, too, lacks absolute precision. Instead, we refer to equivalent viscosity or local microviscosity, representing the viscosity of a homogeneous local environment where the probe response remains consistent.^19−22^ To gain insight into this parameter into subcellular environments, molecular rotors serve as fluorescent probes that undergo internal rotations, resulting in microviscosity-dependent changes in their emissive properties. Figure 1A illustrates classical molecular rotors of practical interest. From there BODIPYs were the most studied molecular systems.^23^ Importantly, the selectivity of fluorescent probes for specific cell regions depends on their chemical properties and the unique microenvironment of each organelle, such as lipid composition, pH, and ion concentration. These probes also demonstrate how selectivity is achieved through both organelle characteristics and probe modifications to enhance membrane targeting.

Transitioning from local microviscosity to membrane tension was necessary due to the uncontrolled and hard-to-calibrate nature of local microviscosity, which depends on excited-state rotation. Membrane tension probes, such as molecular **Flippers**, were proposed as mechanosensing probes designed to visualize physical forces within membranes of living organisms. These probes function by altering their conformational structure through planarization. **Flippers** are twisted out of conjugation due to repulsion polarization of their core in the ground state.^24^ In nonconfining

environments like lipid disordered phases, the endocyclic sulfurs next to the connecting rotatable bond are twisted. In confined environments like lipid ordered phases, the molecule planarizes in the ground state, resulting in red-shifted excitation maxima and longer fluorescence lifetimes. This mechanoprobe is highly sensitive to changes in lipid order and can monitor membrane tension by changing lipid packing through stretching the lipid tails.

Studies measuring the flipper-fluorescence lifetime in isoosmotic, hypoosmotic, and hyperosmotic conditions using MDCK and HeLa cell cultures showed an unexpected increase in **Flipper** lifetime with increasing membrane tension, Figure 1B–C. Then, this has evolved to a new concept called membrane tension probes, which utilizes a ground-state planarization as a new approach to mechanosensitive membrane probes.^25^ The redefinition of local microviscosity represents an evolved concept with broader applications in monitoring subcellular dynamics and addresses the unmet needs of subcellular biology more effectively. While fluorescent molecular rotors remain valuable, their excited-state rotation mechanisms are particularly useful for imaging membrane order.^26,27^

## Adapting Microenvironmental Polarity Probes for Membrane Hydration Monitoring

The study of membrane mechanics in living cells has emerged as a pivotal area of research, driven by the need to understand the physical forces at play within cellular membranes. These forces, while inherently challenging to image directly, manifest through suprastructural changes that can be detected using advanced chemical tools.^28^ Recent advancements have highlighted the utility of solvatochromic probes, which respond to shifts in microenvironmental polarity including hydration and offer promising avenues for imaging membrane order in living cells.^29^ Experimental evidence shows an interplay between membrane tension and hydration, especially in cellular environments where tension changes affect water molecule arrangement within the lipid bilayer. Higher tension can decrease membrane packing and thickness, likely reducing hydration, while lower tension may allow for more hydration. This interplay is crucial when designing hydration probes that can also detect membrane tension. The transition from the concept of membrane local micropolarity to membrane hydration has further refined the application of these probes, allowing for more reliable and practical studies in cell biology.

**Figure 2:**
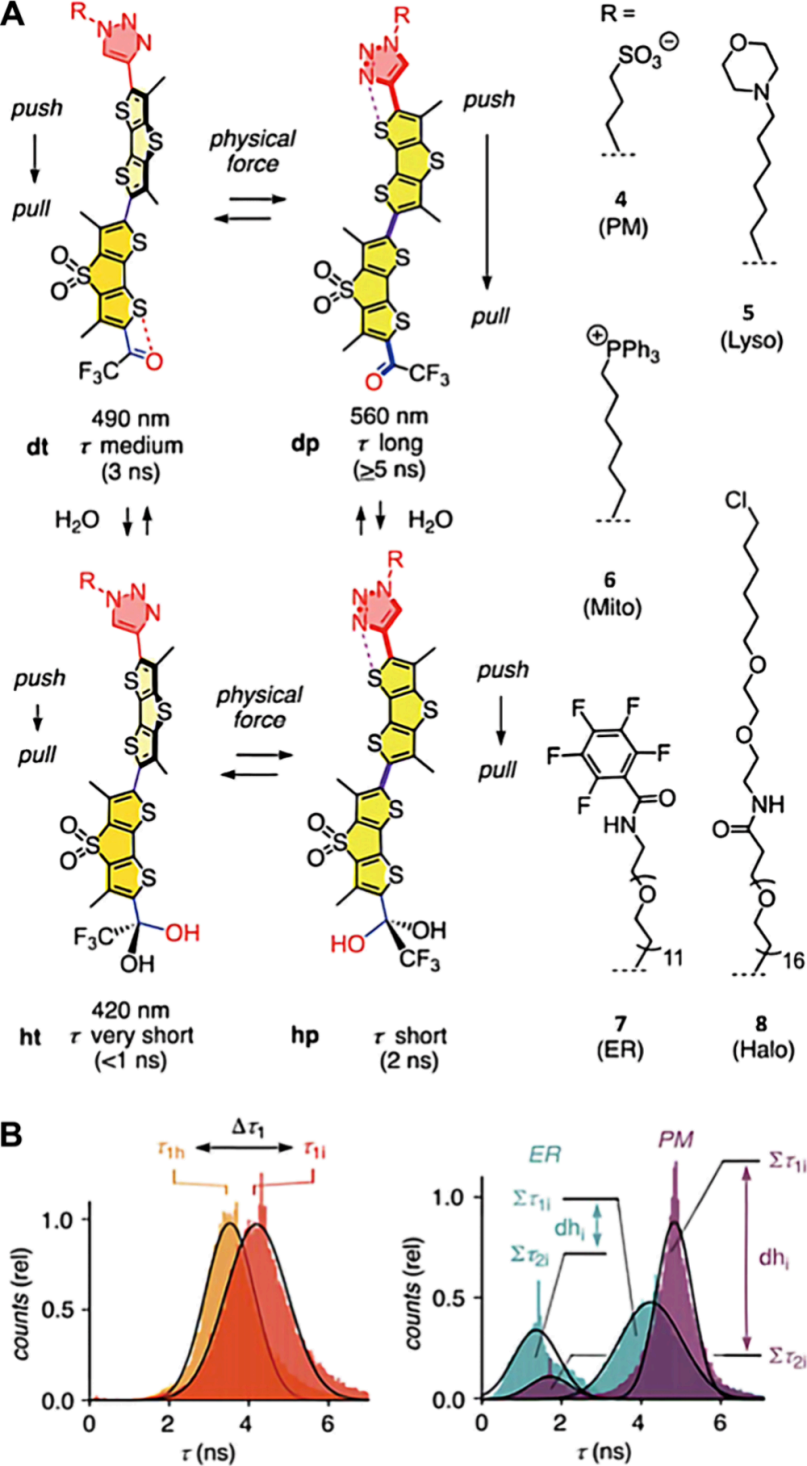
(A) The dual sensing cycle of the published **HydroFlippers**, tested in living cells with indication of excitation maxima and fluorescence lifetimes (B) Distribution of the photon counts (PCs) associated with the τ_1_ component after triexponential reconvolution of lifetime images before and after hyperosmotic shock, showing decreasing lifetimes for τ_1_. The dehydration factor dh_*i*_ is defined as total integrated PCs for τ_1_ (Στ_1_) over Στ_2_ (i.e., dh_*i*_ = area Στ_1i_/area Στ_2i_).

In the realm of fluorescent probes for studying membrane hydration, **HydroFlippers** stand out as innovative tools capable of reporting on both membrane compression and hydration simultaneously, Figure 2A.^30^ These probes are designed with a sensing cycle that couples the mechanical planarization of twisted push–pull fluorophores with dynamic covalent hydration of their exocyclic acceptor. This dual functionality is essential for capturing the interplay between membrane tension and hydration in live cells. Membrane hydration is quantified by analyzing the photon count ratio of hydrated vs dehydrated mechanophores in reconvoluted lifetime frequency histograms, Figure 2B. They identified two distinctly separate populations: τ_1_ (red), τ_2_ (green), and background (τ_3_, blue) for endoplasmic reticulum (ER) and plasma membrane (PM). Thus, the dehydration factor (dh_*i*_) defined by τ_1_/τ_2_ counts, indicated a highly hydrated ER (dh_*i*_ < 2) compared to a weakly hydrated plasma membrane (dh_*i*_ > 6) in HK cells. This sophisticated mechanism allows for precise monitoring of membrane dynamics in various organelles. The application of **HydroFlippers** has revealed intriguing insights: while tension-induced mechanical compression appears relatively consistent across different membrane organelles (MOIs), the response to changes in hydration is highly dependent on the intrinsic order of the MOI.

The subcellular microenvironment is vital for maintaining cellular homeostasis, with hydration playing a crucial role in the organization of lipids within biological membranes. In this context, we introduce **NMP-1**, an organic fluorescent probe specifically designed for selective localization to the plasma membrane in live cells. Utilizing high-resolution confocal microscopy, **NMP-1** allows for real-time

monitoring of hydration and polarity variations, particularly during the formation of apoptotic bodies. To complement this, **CellPol-1**, an analogous polarity-sensitive probe that localizes to the cytosol and nucleoli, was synthesized. This dual-probe system exemplifies a detailed monitoring of subcellular hydration changes, enhancing our understanding of cell microenvironment dynamics. **NMP-1** and **CellPol-1** exhibit high sensitivity as microenvironment indicators, surpassing previous cinnamic-derived dyes. Spectroscopic studies, including UV–vis and fluorescence, show that **NMP-1** is highly responsive to subtle water variations, underscoring its ability to detect membrane hydration, while **CellPol-1** effectively senses polarity. Together, these probes are powerful tools for exploring cellular membrane properties and their response to environmental shifts.

## Redefining Voltage Sensing in Neuron Communication

The development of advanced techniques for imaging electrical activity in excitable cells has significantly deepened our understanding of complex biological systems, such as the brain and heart, across various mammalian species, including humans. The performance of voltage-sensing probes using photoinduced electron transfer mechanisms is often hindered in deep neural tissues, where scattering and low absorption reduce fluorescence signal strength. This limits resolution in complex tissue environments. Two solutions are typically applied: (1) using red-shifted or near-infrared fluorophores to improve penetration and reduce scattering and (2) leveraging advanced imaging methods, such as two- or three-photon excitation microscopy, which enhance sensitivity and enable deeper tissue imaging for precise measurements.

Voltage changes in excitable cells result from the diffusion of ions across cell membranes. At rest, cells maintain a membrane potential of approximately −70 mV, but when neurons are stimulated strongly enough, the membrane depolarizes, with the cytoplasm becoming more positive, reaching potentials around +30 mV. Conversely, when the cytoplasm becomes more negative, the membrane is said to be hyperpolarized.^31^ Despite the small number of ions and molecules involved in these processes, they can induce significant changes in biophysical properties, such as the electric field, posing limitations for some voltage-sensing mechanisms. Another challenge is the extremely thin nature of the plasma membrane, necessitating that voltage-sensitive chromophores either reside within the membrane or maintain direct contact with it. Any conformational changes in these molecules could compromise measurement sensitivity, so it is vital that probes are highly sensitive to detect changes through a membrane only about 5 nm thick. Additionally, the risk of photodamage associated with strong light sources and excited state reactions presents another obstacle.^32^

Traditionally, membrane potential changes have been monitored using invasive techniques like patch-clamp, which involves inserting microelectrodes into intra- and extracellular spaces.^33^ While this method offers high sensitivity, submillisecond temporal responses, and valuable data, it is labor-intensive and typically limited to the study of one or two cells. The use of fluorescent proteins that detect calcium transients, such as the GCaMPs family, provides indirect insights into neuronal activity, enabling the study of dendritic spines and neuronal assemblies.^34^ However, calcium imaging has its limitations, including high costs, slow kinetic responses, and the fact that it requires calibration for optical-electrical conversion, along with the complexities of encoding and expressing the necessary proteins.^35,36^

**Figure 3:**
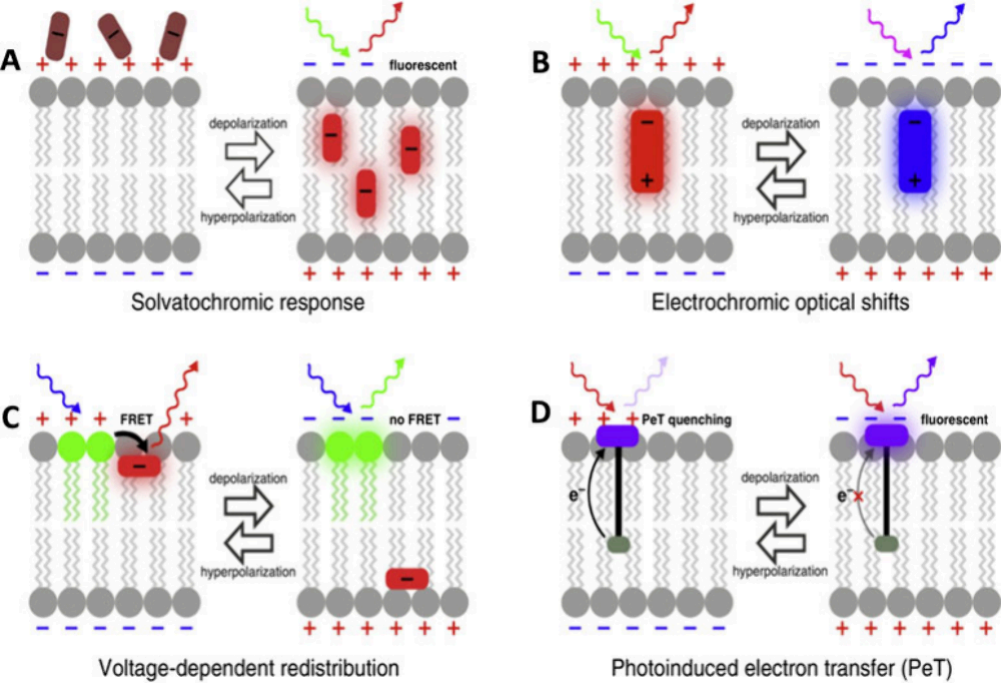
Methods of voltage sensing with small molecules covering the green GFP, λ_exc_ = 488 nm and red Cy5, λ_exc_ = 647 nm channels. (A) Some voltage dyes accumulate in cell membranes in a voltage-dependent manner. (B) Electrochromic dyes interact with the membrane electric field, causing wavelength shifts. (C) Lipophilic anions redistribute across the membrane with changes in potential. (D) Voltage sensing via PeT leverages the field sensitivity of electron transfer in molecular wires.

Recent advances in synthetic organic chemistry have paved the way for the design and synthesis of small-molecule fluorescent probes, allowing for precise tuning of photochemical properties suitable for use in confocal, super-resolution, and multiphoton microscopy. Voltage probes based on small organic molecules leverage various mechanisms, including Förster resonance energy transfer (FRET), aggregation, and electro-optical or electrochromic effects.^32,35^ One promising area of exploration in recent years is the Photoinduced Electron Transfer (PeT) mechanism, which has been used to develop fluorophores with greater sensitivity, higher signal-to-noise ratios, and faster response times.^37,38^ PeT-based probes feature an electron donor–acceptor pair, facilitating an intramolecular transfer that competes with fluorescence emission and is dependent on the surrounding electric field. This principle has led to the development of xanthene-based probes capable of measuring depolarization and hyperpolarization phenomena in various cellular contexts, utilizing both confocal and multiphoton microscopy Figure 3.^39−43^

In the field of neuron communication, the quest to develop fluorescent molecular probes for in-tissue voltage monitoring has faced significant challenges, particularly due to the low tissue penetrability of these fluorophores. The ability to monitor neuronal voltage within tissues is crucial for advancing our understanding of brain function and for the development of new therapies. However, traditional fluorescent probes have been limited by their poor penetration into deep tissues, making them less effective for *in vivo* applications.

In fact, there remains an unmet need for fluorescent molecular probes capable of effectively monitoring neuronal voltage within tissues, primarily due to the insufficient tissue penetration of these fluorophores.^44^ In this vein, recent advancements in optogenetics have offered promising alternatives to overcome this limitation. Optogenetics, a technique that uses light to control cells within living tissue, has been pivotal in allowing researchers to manipulate neuronal activity with high precision. Nevertheless, the reliance on visible light (e.g., blue or yellow) presents a major drawback due to its limited tissue penetration, often necessitating the use of invasive fiber-optic probes. These probes can cause tissue damage and induce unwanted inflammation, complicating their use in sensitive applications.^45^

The emergence of wireless optogenetic tools that respond to near-infrared (NIR) light has opened new avenues for less invasive neuronal control. NIR light, with its superior tissue penetration compared to visible light, minimizes the risk of tissue damage. There are primarily two types of NIR-activatable optogenetic tools: one that uses lanthanide-doped up conversion nanoparticles to convert NIR light to visible light, modulating classical opsin-expressing neurons;^46^ and another that couples with an NIR absorber to generate heat, thereby activating thermosensitive proteins.^47^ These innovative approaches allow for the remote control of cellular signaling pathways, potentially leading to more effective therapies for neurological disorders and other diseases.

The integration of NIR-activatable optogenetic tools into the study of neuronal communication represents a significant leap forward.^48^ By overcoming the penetrability issue that has long hindered the development of fluorescent probes for in-tissue voltage monitoring, these tools offer a powerful new method for studying and controlling neuronal activity in a minimally invasive manner. As research in this field advances, it has the potential to transform approaches to treating complex diseases and deepen our understanding of the brain communication networks.

## Reshaping Aggregation-Induced Emission (AIE) for Membrane Partitioning Probes

The quest for effective membrane partitioning probes has seen significant advancements, particularly through innovative approaches like the coaggregation technique pioneered by B. Z. Tang and colleagues.^49,50^ This method has set a benchmark for fluorescent probe development, allowing precise cellular membrane tracking. However, challenges remain in ensuring probe specificity and functionality over extended periods, especially for plasma membrane (PM) tracking.

**Figure 4:**
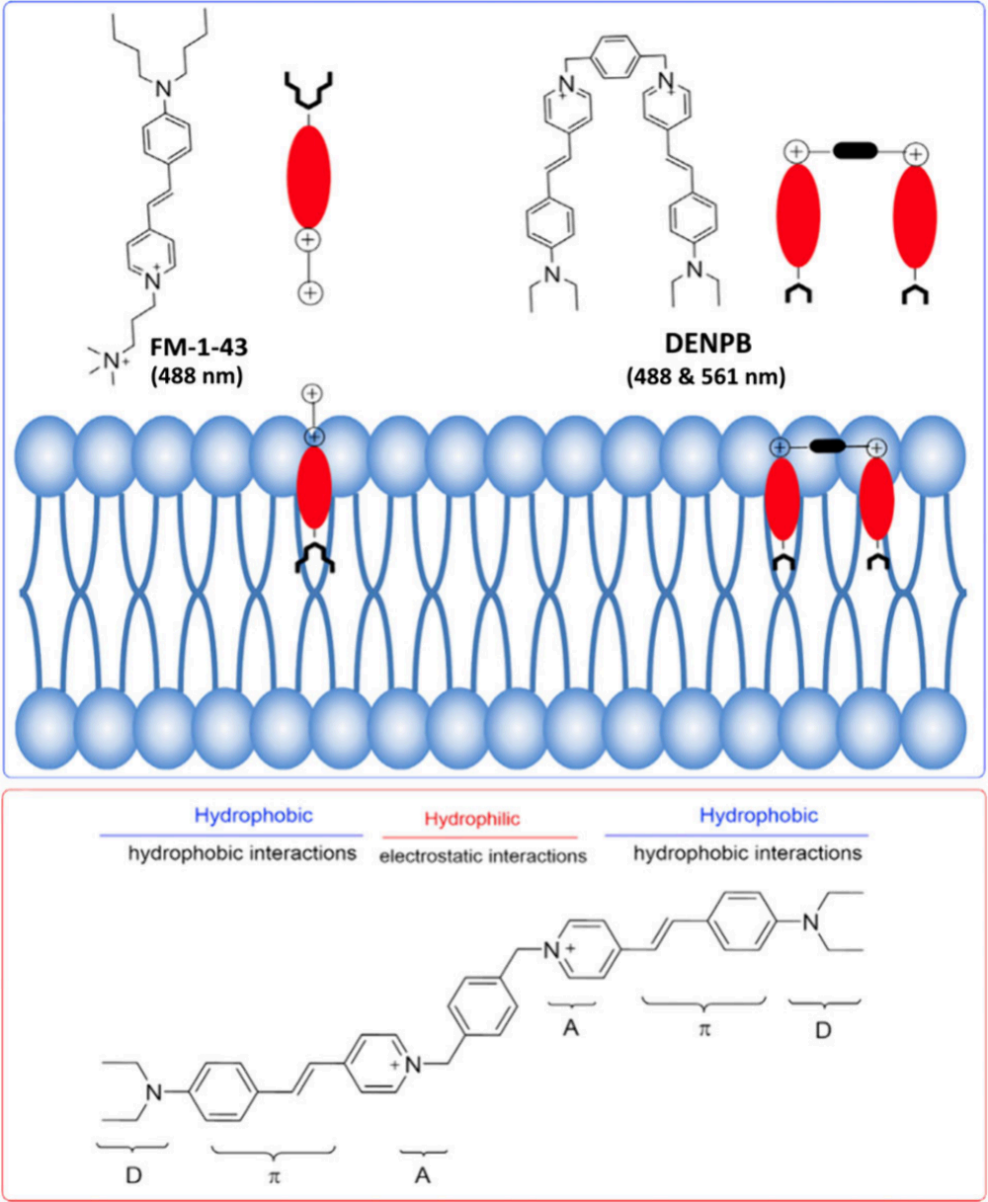
Schematic illustration of the structural characteristics of **FM1–43** and **DENPB**, with customized wavelengths indicated and their interactions with the phospholipid bilayer in the PM, also showing the chemical structure and design strategy of **DENPB**.

Unlike conventional fluorescent probes, AIE probes enhance luminescence upon aggregation while minimizing nonradiative decay, which allows for extended imaging sessions with minimal signal loss. However, despite this high photostability, AIE probes can still undergo photobleaching during prolonged imaging under high-intensity excitation. This balance between resilience and limitations is crucial for applications requiring durable, high-intensity imaging. To address this, recent research introduced **DENPB**, an amphipathic aggregation-induced emission (AIE)-active far-red fluorescent probe, optimized for long-term PM imaging. In live cancer cells, **DENPB** activates upon PM insertion, showing sustained retention and enabling PM tracking for up to 6 h without significant internalization, as shown in Figure 4.

**DENPB** is particularly notable for its selective staining capability, as it specifically targets the PM of live cancer cells, including HeLa, HepG2, MDA-MB-231, and MCF-7, while showing no signal in live noncancer cells like RAW 264.7, NIH 3T3, and HFF. Additionally, **DENPB** distinguishes between paraformaldehyde-fixed cancer cells and noncancer cells by producing distinct signal patterns. As a reliable and selective tool for monitoring plasma membrane dynamics, **DENPB** and similar probes could significantly enhance our understanding of membrane biology and improve early cancer detection and diagnosis.^51^

Similarly, the Nernst partition law has been leveraged for the selective targeting of lipid droplets. Building on the principle of aggregation state and the Nernst partition law, Jiménez-Sánchez et al. devised a strategy for selective targeting of LDs. Their approach involved recording and monitoring LDs in one emission color (monomeric species), while the surrounding aqueous environment was captured in a different response channel.^52^ This strategy draws parallels to the widely recognized Nernstian mechanism used for mitochondrial probes, where membrane potential facilitates targeting through electrostatic interactions.

**Figure 5:**
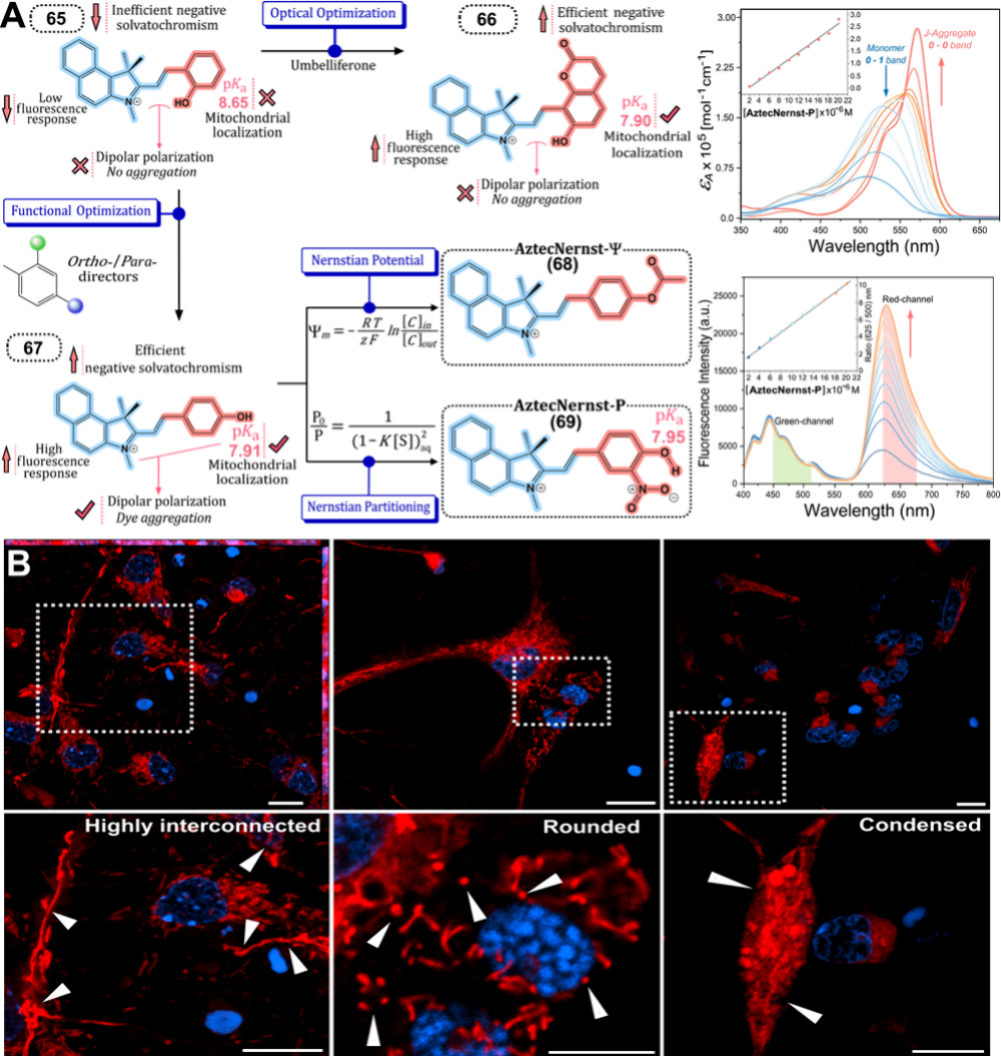
(A) **AztecNernst** fluorophores approach using a classic extension of a benz[*e*]indolinium scaffold with an *ortho*-phenolic (**65**), 4-methylumbelliferone (**66**), and *para*-fenolic (**67**) substitution to get the optimized Nernst potential **AztecNernst-Ψ** (**68**) and **AztecNernst-P** (**69**). UV–vis absorption and fluorescence spectra of **69** from 2 to 20 μM increased concentration. (B) Mitochondrial dynamics changes in primary cortical neurons under normoxia and oxygen glucose deprivation (OGD). Reproduced from Ordóñez-Hernández et al., 2024 with permission from © Royal Society of Chemistry, 2024.^53^

Their study revealed that the equilibrium distribution between two immiscible liquid phases—cytosolic and LD compartments—could act as the driving force for accumulating the molecular probe into the LDs. This accumulation

occurs via a partitioning scenario involving monomeric (highly lipophilic phase) and self-associated (aqueous phase) species. The Nernst partition coefficient in LDs is defined as the equilibrium constant for the distribution of species between the LD phase and the surrounding cytosolic phase, shedding light on the intricate interplay between molecular properties and cellular compartments in targeted imaging applications. These advancements in membrane partitioning fluorescent probes underscore the potential for enhanced clarity and precision in exploring cellular dynamics, offering promising avenues for the future of bioimaging, Figure 5.

## Reassessing the Roles of Specific Reactive Oxygen Species in Physiology

Reactive oxygen species (ROS) encompass a broad array of oxidants derived from molecular oxygen. These species are part of a larger group that includes reactive nitrogen, sulfur, carbon, selenium, electrophile, and halogen (RHS) species, all capable of undergoing redox (reduction–oxidation) reactions. They induce oxidative modifications on biological macromolecules, playing crucial roles in redox signaling and cellular functions. However, at supraphysiological concentrations, ROS can react indiscriminately with proteins, lipids, nucleic acids, and carbohydrates, generating other reactive species that may have harmful effects.^54,55^

The new generation of ROS probes incorporates selective design features, such as tailored chemical groups that specifically react with target oxidants. For example, James, et.al. reported a triflate group that selectively reacts with superoxide, while its phenolic product detects peroxynitrite. These probes can also use spatial or temporal activation mechanisms to target specific cellular conditions. Calibration in biological contexts helps reduce false positives, ensuring probes respond accurately. Using complementary probes for various oxidants will allow a broader understanding of oxidative stress with minimized nonspecific signals.^56^

Small-molecule caged fluorophores are less susceptible to artifacts and offer improved specificity. Currently, there are no genetically encoded probes specifically for superoxide (O_2_^•–^).^57−60^ The most validated fluorescent probe for O_2_^•–^ is hydroethidine, and its mitochondrially targeted version, **MitoSOX**. However, an increase in fluorescence from these probes does not necessarily indicate O_2_^•–^ production, as hydroethidine can nonspecifically oxidize to form fluorescent ethidium.^61^ Newer, although not yet widely adopted, fluorescent probes based on **NeoD** show greater selectivity and physiological relevance as they do not intercalate with DNA, unlike ethidium compounds.^62^

Alternately, fluorescent probes targeting biomarkers, such as enzymes, proteins, and DNA, have also become essential tools for visualizing and tracking specific cellular processes, enabling researchers to detect and monitor disease-associated molecules with high precision. For example, the significance of fluorescence probes in detecting biomarkers such as cyclooxygenase-2 (COX-2), a critical enzyme overexpressed in various cancers.^63^ Recent advancements include the development of probes such as NP-C6-CXB and NB-C6-CCB, which demonstrate strong fluorescence selectivity for COX-2 in cancer cells, enabling clear differentiation between cancerous and normal cells. These probes are effective due to their selective binding and subcellular targeting, enhancing imaging precision.^64,65^

## Conclusions

As an overarching view, redefining molecular probes for monitoring subcellular environments by focusing on biologically relevant physicochemical parameters, represents a bridge for exploring applications in fluorophore chemistry. While traditional probes have offered valuable insights, their limitations in capturing the dynamic and complex nature of physiological conditions are clear. By advancing the design and application of fluorescent probes, we can more accurately reflect the intricate biophysical properties of living systems, particularly in real-time and under stress or disease conditions. This new paradigm not only enhances our understanding of cellular dynamics but also paves the way for more precise diagnostic tools and therapeutic interventions. Future efforts should focus on developing robust, selective, and adaptable probes that address the unmet challenges of subcellular biology, ensuring broader applications in biomedical research and clinical settings. Through these innovations, we aim to inspire a new era of biosensing that bridges the gap between research and clinical practice, ultimately advancing personalized medicine and real-time physiological monitoring.
